# Alfalfa-containing diets alter luminal microbiota structure and short chain fatty acid sensing in the caecal mucosa of pigs

**DOI:** 10.1186/s40104-017-0216-y

**Published:** 2018-01-09

**Authors:** Jiawei Wang, Chunfu Qin, Ting He, Kai Qiu, Wenjuan Sun, Xin Zhang, Ning Jiao, Weiyun Zhu, Jingdong Yin

**Affiliations:** 1 State Key Laboratory of Animal Nutrition, College of Animal Science and Technology, China Agricultural University, No. 2 Yuanmingyuan West Road, Beijing, 100193 China; 2 Jiangsu Key Laboratory of Gastrointestinal Nutrition and Animal Health, College of Animal Science and Technology, Nanjing Agricultural University, No.1 Weigang, Nanjing, 210095 China; 3 College of Biotechnology and Food Science, Hebei Normal University for Nationalities, Chengde, 067000 China

**Keywords:** Alfalfa meal, Growing pigs, Insoluble fiber, Microbiota, SCFA

## Abstract

**Background:**

Pork produced by outdoor-reared pigs raised mostly on alfalfa pastures attracts increasing population of consumer from most of the world. In China, pigs were raised with alfalfa-containing diets to seek for good quality pork. However, the influence of dietary alfalfa involving high level of insoluble dietary fiber (IDF) on pig intestinal luminal microbiota composition remains unclear. The objective of this study was to investigate the effects of alfalfa on luminal microbiota and short chain fatty acids (SCFA) production, and gene expressions involved in SCFA sensing, transporting and absorbing in pig caecal mucosa.

**Results:**

Twenty-four growing pigs were randomly allotted to four diets containing 0%, 5%, 10% and 15% alfalfa meal for a 28-d experiment. Ingestion of alfalfa meal-contained diets significantly increased the ratio of body weight gain to feed consumption. Illumina MiSeq sequencing of the V3 region of the 16S rRNA genes showed that alfalfa-containing diet significantly decreased the relative abundance of genera *Turicibacter*, *Acidiphilium*, *Paracoccus*, *Propionibacterium*, *Corynebacterium*, *Pseudomonas*, *Acinetobacter*, and *Staphylococcus*, and increased the relative abundance of genera *Lachnospira*, *Marvinbryantia*, and *Desulfovibrio* in the caecal digesta. Butyrate concentration was significantly increased in the hindgut by the supplementation of alfalfa meal in diets. The mRNA gene expressions of *FFAR3*, *SMCT1*, *MCT1*, *PYY*, and *GCG* were significantly increased in the caecal mucosa of pigs fed alfalfa meal.

**Conclusions:**

Our results suggested that alfalfa-containing diet has exerted significant impacts on caecal microbiota composition, butyrate concentration and significantly upregulated mRNA expression of host caecal mucosal genes involved in SCFA sensing and absorption as well as regulation of satiety.

**Electronic supplementary material:**

The online version of this article (10.1186/s40104-017-0216-y) contains supplementary material, which is available to authorized users.

## Background

It has been well demonstrated that dietary fiber (DF) plays a positive role in maintaining diversity of gut microbial community and subsequent intestinal health in humans and pigs [1, 2]. A high-fiber diet can also increase the activity of fiber-degrading related bacteria in the large intestine of growing pigs [3, 4]. These increased amount of cellulolytic bacteria favor the colonization and growth of some beneficial bacteria, meanwhile, decrease the harmful ones, which is beneficial to intestinal health and supported as a prebiotic effect [5]. Previous studies mainly focused on the beneficial effects of soluble DF or resistant starch on intestinal microbial structure and metabolites, as well as intestinal mucosal physiological reactions [6]. However, there are few reports concerning the effects of insoluble dietary fiber (IDF).

Alfalfa DF is mainly composed of IDF, which includes cellulose, xylans and lignin, and accounts for more than 90% of the total DF content of alfalfa [7, 8]. Natural pork is highly appreciated by customs in Europe and North America. It is produced by pigs that are ordinarily reared on alfalfa pastures in outdoor rearing system. Similarly, alfalfa is also used as green fodder in traditional-style pig feeding system in China for better quality pork. Along with increasing demand of these styles of pork, the effect of alfalfa on gut microbial community of pigs rapidly drew more attention.

Acetate, propionate and butyrate are the main microbial fermentation products of DF in the caecum and colon of pigs, which account for more than 95% of the total SCFA content [8]. These SCFA have been shown to benefit host health and energy metabolism [9, 10]. It has been known that SCFA are transported actively across the apical membranes via SCFA transporters sodium-coupled monocarboxylate transporter 1 (SMCT1) and monocarboxylate transpoter 1 (MCT1) in caecal and colonic epithelial cells [11, 12]. It has been documented that among SCFAs, increasing concentration of butyrate could raise mRNA expression of gene *SMCT1* and *MCT1* in caecal and colonic mucosa [13]. Luminal butyrate could also be transported into colonic epithelial cells and has access to histone deacetylases via its high-affinity transporter MCT1, which is critical for the process of butyrate to inhibit histone deacetylases [14]. SCFA concentrations are also sensed by SCFA receptors free fatty acid receptor (FFAR) 2 and FFAR3 [15, 16]. FFAR2 and FFAR3 can also stimulate gut hormones peptide YY (PYY) and glucagon-like peptide 1 (GLP-1) released by enteroendocrine L-cells [17–19]. PYY positively regulates satiety, and increased *PYY* expression can reinforce the sensitivity of insulin, reduce feed intake and contribute to maintenance of body energy balance [19, 20]. GLP-1 can indirectly modulate blood glucose through increasing the secretion of insulin and reducing the secretion of glucagon by the pancreas [21]. Presently, it is not clear whether the physiological process composed by the expression of above genes in the intestinal mucosa can be affected by the ingestion of alfalfa-containing diets.

Therefore, the current study was carried out to investigate the effects of alfalfa typically contained abundant IDF on luminal microbiota composition, SCFA output, as well as the mRNA expression of host SCFA sensing genes which is vital to pig health.

## Methods

### Experimental diets, animals, and feeding

The experimental diets were formulated to provide the equal amount of net energy (2475 kcal/kg) and the standard ileal digestible amino acids, which met the nutrient requirements for growing pigs recommended by NRC (2012). Table 1 shows the dietary ingredients and nutrient composition of the experimental diets. None of antibiotic additives was included in experimental diets. The experimental diets were sampled and stored at -20 °C until analysis. Determined nutrient composition and non-starch polysaccharides contents of alfalfa meal used in this experiment were showed in Additional file 1: Table S1.

**Table 1:** Ingredients and nutrient composition of the experimental diets (as-fed basis)

Item	Level of alfalfa meal, %			
	0	5	10	15
Ingredient, %				
Corn	71.10	64.97	58.00	52.54
Soybean meal	25.00	24.60	25.00	24.00
Alfalfa meal^a^	0.00	5.00	10.00	15.00
Soybean oil	0.52	2.20	3.99	5.58
Limestone	1.16	1.00	0.88	0.72
Dicalcium phosphate	0.70	0.70	0.65	0.65
Sodium chloride	0.30	0.30	0.30	0.30
L-Lysine	0.24	0.24	0.21	0.22
L-Threonine	0.08	0.08	0.06	0.07
L-Tryptophan	0.02	0.02	0.02	0.02
DL-Methionine	0.07	0.08	0.08	0.09
Vitamin-mineral premix^b^	0.81	0.81	0.81	0.81
Energy and Nutrient composition^c^				
DM, %	88.25	88.71	89.24	89.75
CP, %	16.93	17.54	17.89	17.61
NDF, %	12.92	14.71	16.10	18.21
ADF, %	5.88	6.21	7.45	8.44
Lys, %	1.12	1.16	1.11	1.17
Met + Cys, %	0.60	0.64	0.65	0.71
Try, %	0.18	0.20	0.19	0.18
Thr, %	0.76	0.80	0.77	0.80
NE, kcal/kg	2475	2475	2475	2475
Soluble dietary fiber, %	1.82	1.51	2.04	2.18
Insoluble dietary fiber, %	12.66	13.02	14.82	16.80
Total dietary fiber, %	14.48	14.52	16.86	18.98

^a^Alfalfa meal: 91.3% DM, 16.5% CP, 50.6% NDF, 34.3% ADF, 12.5% crude ash

^b^This vitamin-mineral premix supplied per kg diet as follows: vitamin A as retinyl acetate, 4000 IU; vitamin D_3_ as cholecalciferol, 1000 IU, vitamin E as DL-alpha tocopheryl acetate, 10 IU; vitamin K_3_ as menadione nicotinamide bisulfite, 1.25 mg; thiamine as thiamine mononitrate, 0.5 mg; riboflavin, 2.1 mg; pyridoxine as pyridoxine hydrocloride, 1 mg; vitamin B_12_, 0.007 mg; D-pantothenic acid as D-calcium pantothenate, 6 mg; niacin as nicotinamide and nicotinic acid, 12 mg; folic acid, 0.25 mg; biotin, 0.02 mg; Cu, 10 mg as copper sulfate; Fe, 75 mg as iron sulfate; I, 0.025 mg as potassium iodate; Mn, 10 mg as manganese sulfate; Se, 0.02 mg as sodium selenite; Zn, 45 mg as zinc oxide; choline chloride (50%), 1.2 g; sweeteners, 0.1 g; feed flavor, 0.1 g; phytase, 0.1 g

^c^The NE is calculated, whereas all other values are analyzed

A total of 24 castrated male pigs (Duroc × Landrace × Yorkshire) with initial body weight (BW) of 24.8 ± 0.7 kg were housed individually in metabolism crates (1.4 m × 0.7 m × 0.6 m) with ambient temperature maintained at 25 ± 2 °C for the duration of the experiment. Pigs were divided into four groups in a completely randomized design to receive one of four experimental diets supplemented with 0%, 5%, 10% and 15% alfalfa meal, with 6 replications per treatment and one pig per replication. Pigs were allowed to ad libitum access to feed and water for a period of 28-d experiment.

### Harvest and sample collection

At the end of the trial, pigs were electronically stunned and exsanguinated, after which the abdominal cavity was opened and the gastrointestinal tract was ligated and removed from the enterocoelia. The caecal and colonic digesta were collected individually for subsequent quantification of SCFA concentrations. In addition, four representative caecal digesta samples were selected separately from pigs fed the control diet (hereinafter referred to as CAE0) and pigs fed 15% dietary alfalfa meal (hereinafter referred to as CAE15) for subsequent analysis of microbiota composition. A small piece from the caecal and colonic segments was excised and rinsed in 0.9% saline solution for subsequent RNA isolation. All the samples were immediately flash frozen in liquid nitrogen and stored at −80 °C until further analysis.

### Caecal and colonic SCFA quantification

Concentrations of acetate, propionate and butyrate in caecal and colonic digesta of the pigs were analyzed using Dionex ICS-3000 Ion Chromatography System (Dionex Corporation, Sunnyvale, CA) as described previously [22].

### Bacterial DNA extraction, PCR amplification, and Illumina MiSeq sequencing

The total genomic DNA was extracted from caecal digesta using Power Soil DNA Isolation Kit (12888–50; Mobio Technologies Inc., Vancouver, BC) following the manufacture recommendations. The quality of extracted DNA was checked by 1% agarose gel electrophoresis and spectrophotometry (optical density at 260 nm/280 nm ratio). All extracted DNA samples were stored at −20 °C for further analysis. The V3 hypervariable region of the bacterial rRNA gene was amplified using the barcoded fusion primers (forward primer: 338-CCTACGGGAGGCAGCAG-355, reverse primer: 502-ATTACCGCGGCTGCTGG-518). PCR reactions were performed in triplicate 25 μL mixture containing 2.5 μL of 10 × pyrobest buffer, 2 μL of 2.5 mmol/L dNTPs, 1 μL of each primer (10 μmol/L), 0.4 IU of TransStart Fastpfu DNA Polymerase (AP221–02; TransGen Biotech Co., Ltd. Beijing, China), and 15 ng of template DNA. The PCR programs were 94 °C for 5 min; 94 °C for 30 s, 48 °C for 30 s and 72 °C for 30 s, repeat for 25 cycles; 72 °C for 10 min. Amplicons were extracted from 2% agarose gels and purified using the AxyPrep DNA Gel Extraction Kit (Axygen Biosciences, Union City, CA) according to the manufacturer’s instructions and quantified using QuantiFluor™-ST (Promega Inc., Madison, WI). Purified amplicons were pooled in equimolar and paired-end sequenced (2 × 150) on an Illumina MiSeq platform according to the standard protocols.

### Bioinformatic analysis

The extraction of high-quality sequences was firstly performed with the QIIME package (version 1.8). The raw sequences were filtered according to the barcode and primer sequences, and the resulting sequences were further screened and filtered for quality. Sequences with an average phred score lower than 25, containing ambiguous bases, homopolymer run exceeds 6, having mismatches in primers, or sequence length shorter than 100 bp were removed. Sequences that overlap longer than 10 bp and without any mismatch were assembled according to their overlap. The unique sequence set was classified into operational taxonomic units (OTUs) under the threshold of 97% identity using UCLUST (version 1.2.22). Chimeric sequences were identified and removed using USERCH (version 8.1.1861). The taxonomy of each 16S rRNA gene sequence was analyzed using the Uclust Consensus Taxon Assigner against the SILVA (Release 119) 16S rRNA database with a confidence threshold of 90%. The ACE and Chao1 community richness estimator, the Shannon and Simpson community diversity indices were calculated with the MOTHUR program.

### RNA isolation, cDNA synthesis, and quantitative real-time PCR

Total RNA was isolated from caecal and colonic tissue by using RNeasy Plus Universal Kit (73404; QIAGEN, Venlo, Netherlands) according to the manufacturer instructions. Concentration and purity of RNA samples were determined on a NanoDrop ND-1000 spectrophotometer (Thermo Scientific, Waltham, MA).

Single-stranded cDNA was synthesized from 1 μg of total RNA by using the First Strand cDNA Synthesis Kit (CW0741A; CW Biotech Inc., Beijing, China) according to the manufacturer instructions. Quantitative real-time PCR was carried out with the use of Takara real-time PCR Kit (RR096A; Takara Bio Inc., Tokyo, Japan). The reaction system of quantitative real-time PCR was 10 μL including 5 μL SYBR premix, 1 μL cDNA, 0.2 μL forward and reverse primers (10 mmol/L), 0.2 μL ROX and 3.4 μL double-distilled water. Quantitative real-time PCR was performed on Applied Biosystems 7500 Real-Time PCR System (Life Technologies, Foster City, CA). The expression of the genes was calculated relative to the expression of the housekeeping gene *β-actin* with the formula 2^-ΔΔCt^ [23]. Amplification of specific transcripts was confirmed by melting curve profiles at the end of each PCR. The primer sequences were listed in Additional file 1: Table S2.

### Chemical analysis

The diets were analyzed for DM and CP according to AOAC (2007) [24]. Amino acid contents of feedstuff and complete diets were determined using Ion-Exchange Chromatography by an amino acid Analyzer (L8800; Hitachi Ltd., Tokyo, Japan). Neutral detergent fiber and acid detergent fiber were determined by the procedures of Van Soest et al. [25]. Dietary fiber was analyzed by the enzymatic-chemical method as described by Bach Knudsen [7].

### Statistical analysis

Data were analyzed using the PROC GLM of SAS, version 9.3 (SAS Institute, Cary, NC) as a completely randomized design with the fixed effect of diet was used to test for differences in animal performance and SCFA concentration. The effects of ingestion of alfalfa meal containing diet on the microbial richness, diversity and caecal mucosa gene expression were tested for significance using Student’s t-test. Results of SCFA concentrations and mRNA expression were performed using GraphPad Prism, version 6.0. For comparing differences in microbiota composition, the relative abundance at phylum and genus levels in the caecum and colon was processed by non-parametric Mann-Whitney *U* test with corrected *P* value. The differences were considered significant when *P* < 0.05 and a trend when the *P* value was between 0.05 and 0.10.

## Results

### Performance

Final BW of pigs as well as average daily gain (ADG) was similar among the dietary treatments (Table 2). The average daily feed intake (ADFI) tended to be decreased by the diet containing 15% alfalfa meal relative to the control diet (linear, *P* = 0.08). The diets supplemented with 5, 10 and 15% of alfalfa meal increased the gain:feed (G:F) ratio compared with the control diet (linear, *P* = 0.02) while no difference was observed among the three alfalfa meal contained diets (both linear and quadratic, *P* > 0.05).

**Table 2:** Effects of alfalfa meal on the growth performance of growing pigs (*n* = 6)

Item	Level of alfalfa meal, %				SEM	*P*-value	
	0	5	10	15		Linear	Quadratic
Initial BW, kg	25.13	24.70	24.72	24.58	0.65	0.56	0.46
Final BW, kg	46.52	46.48	46.58	46.02	0.91	0.74	0.77
ADG, g/d	713	726	729	714	14.74	0.91	0.36
ADFI, g/d	1439	1407	1410	1369	24.82	0.08	0.85
G:F	0.50^a^	0.52^b^	0.52^b^	0.52^b^	0.01	0.02	0.18

^a,b^Mean values with different letters differ (*P* < 0.05)

### Short-chain fatty acid concentrations of hindgut digesta

**Fig. 1:**
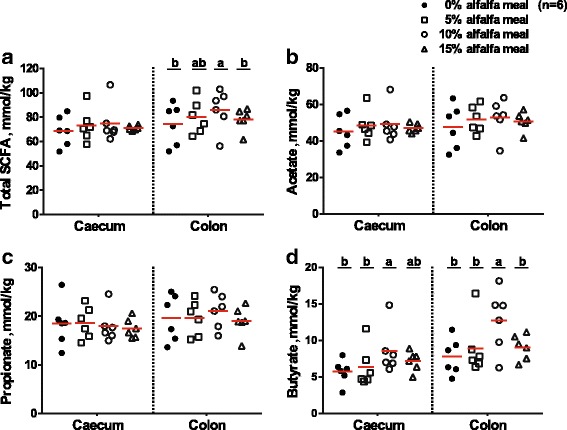
SCFA concentrations (mmol/kg wet digesta) measured in the caecum and colon of pigs fed diets with 0%, 5%, 10% and 15% levels of alfalfa meal (*n* = 6). **a** Total SCFA, **b** acetate, **c** propionate, and (**d**) butyrate. Values are presented as means (red line). ^a,b^Mean values with different letters differ (*P* < 0.05). Each pig sample has an individual symbol. Solid circle, hollow square, hollow circle, and hollow triangle represent pigs fed 0%, 5%, 10% and 15% level of alfalfa meal, respectively

Total SCFA, acetate and propionate concentrations in the caecal digesta were not altered by the dietary treatments (*P* > 0.05) (Fig. 1). However, butyrate concentration was increased by the increased supplementation of alfalfa meal in the diets (*P* < 0.01). Colonic acetate and propionate concentrations were not influenced by increasing levels of alfalfa meal as well (*P* > 0.05). Total SCFA and butyrate concentrations in the colonic digesta was increased significantly (*P* < 0.05). Notably, the butyrate concentration in the caecal and colonic digesta was significantly increased by the supplementation of 10% alfalfa meal in the diet (*P* < 0.05).

### Bacterial richness and diversity of caecal microflora

Illumina Miseq sequencing on the V3 region of bacterial 16S rRNA gene from caecal and colonic digesta samples generated a total of 504,107 high-quality sequences, with an average of 63,013 sequences per sample. All of OTUs were delineated at 97% species similarity level, 8557 OTUs were obtained from caecal digesta samples, with an average of 1069 OTUs per sample. Additionally, Chao1 index of CAE15 group is significantly lower than CAE0 group (*P* < 0.05) while Alatalo index is significantly higher in the CAE15 group (*P* < 0.05) (Table 3). However, there were no statistically significant differences in ACE index, Shannon index or Simpson’s index between CAE0 and CAE15.

**Table 3:** Diversity estimation of the 16S rRNA gene libraries from microbiota in the caecum of pigs fed diets with 0% (CAE0) and 15% (CAE15) levels of alfalfa meal (*n* = 4)

Group	Chao1	Simpson	Shannon	Alatalo
CAE0	1337.9 ± 49.8	0.062 ± 0.012	6.00 ± 0.16	0.712 ± 0.098
CAE15	1127.7 ± 31.5	0.037 ± 0.005	6.17 ± 0.14	1.011 ± 0.036
*P*-value	0.012	0.126	0.448	0.029

### The overall bacterial community structure in caecum

**Fig. 2:**
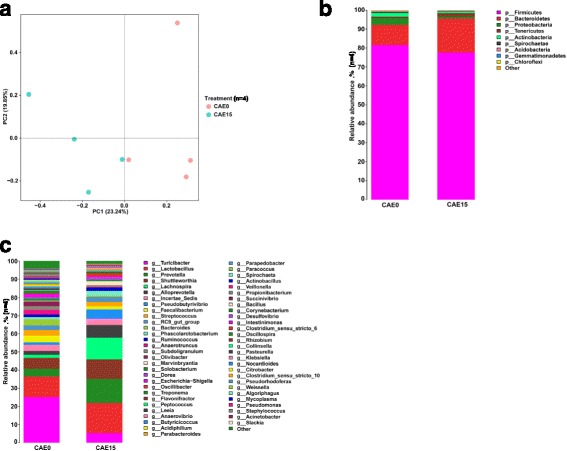
Caecal microbiota composition of pigs fed diets with 0% (CAE0) or 15% (CAE15) level of alfalfa meal (*n* = 4). Principal component analysis evaluated the overall structural changes of the gut microbiota between the CAE0 and CAE15 group (**a**). Relative abundances of samples evaluated at the phylum (**b**) and genus (**c**) levels

Principal component analysis (PCA) revealed that the gut microbiota changed in the caecal digesta in response to the supplementation of 15% alfalfa meal in the diet (Fig. 2a). The differences between the CAE0 and CAE15 group were mainly observed along PC1, which accounted for the largest proportion (23.24%) of the total variation. At the phylum level, Firmicutes was the most predominant phylum whose relative abundance was more than 78%, followed by Bacteroidetes and Tenericutes in the CAE15 group (Fig. 2b). Additionally, the diet supplemented with alfalfa meal significantly decreased the relative abundance of Proteobacteria, Actinobacteria, Gemmatimonadetes and Chloroflexi (*P* < 0.05), and increased the relative abundance of Tenericutes (*P* < 0.05). At the genus level, *Lactobacillus*, *Prevotella*, *Shuttleworthia* and *Turicibacter* were the dominant genera in the CAE15 group (Fig. 2c). The diet with alfalfa meal significantly decreased the relative abundance of *Turicibacter*, *Acidiphilium*, *Paracoccus*, *Propionibacterium*, *Corynebacterium*, *Pseudomonas*, *Acinetobacter*, and *Staphylococcus* (*P* < 0.05). Moreover, the addition of alfalfa meal significantly increased the relative abundance of *Lachnospira*, *Marvinbryantia*, and *Desulfovibrio* in the caecum (*P* < 0.05). In addition, these results suggested that the diets containing alfalfa meal has changed the luminal microbiota structure in the caecum.

### Relatively mRNA expression of genes

**Fig. 3:**
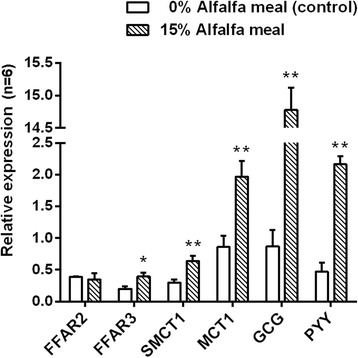
Relative expression of host genes involved in SCFA sensing, uptake and satiety regulation in caecal mucosa of pigs fed diets with 0% (control) or 15% level of alfalfa meal as determined by quantitative real-time PCR (n = 6). FFAR, free fatty acid receptor; SMCT1, sodium-coupled monocarboxylate transporter 1; MCT1, monocarboxylate transpoter 1; GCG, proglucagon; PYY, peptide YY. All data were normalized to an internal *β-actin* mRNA control. Values are presented as means ± SEMs. Mean values differ within the intestinal segment, *P < 0.05, **P < 0.01

In the present study, we determined the mRNA expression of several genes which are involved in the SCFA sensing and absorption in the caecal mucosal enterocytes, including SCFA receptor genes *FFAR2* and *FFAR3*, SCFA transporter genes *SMCT1*, *MCT1* (Fig. 3). In addition, we also investigated the expression pattern of PYY and proglucagon (GCG, the precursor for GLP-1 and GLP-2), which are involved in the regulation of satiety and insulin sensitivity in the caecal mucosa.

In the caecum mucosa, the mRNA expression of *FFAR3* (*P* < 0.05), *SMCT1* (*P* < 0.01) and *MCT1* (*P* < 0.01) was increased in pigs offered 15% dietary alfalfa meal. Moreover, the mRNA expression of *PYY* and *GCG* was significantly increased by the supplementation of 15% alfalfa meal in the diet (*P* < 0.01).

## Discussion

It has been well shown that DF is beneficial to intestinal health in humans and animals [26, 27]. However, among DF, the effects of IDF on the gut microbiota and its metabolites and mucosa relevant gene mRNA expression of pigs are still unclear. Insoluble DF accounts for more than 90% of total amount of DF in alfalfa meal. Therefore, in the present study, we investigated the effect of alfalfa-derived IDF by adding gradient alfalfa meal on the balanced-basal diet. We balanced SID amino acid content, net energy level and fatty acid profile among four dietary treatments. Because major variation of dietary fiber contents among treatments derived from IDF (Table 1), we deduced that differences observed in gut microbiota, SCFA, and gene expression may be due to the specific effect of alfalfa-derived IDF. The ADG and ADFI of pigs were not affected but the G:F was significantly increased by the addition of alfalfa meal, which was in agreement with previous studies [28–30].

Concentrations of SCFA are important indicators for assessment of hindgut fermentation intension [31]. Previous study has shown that IDF has a great effect on fermentation and generation and absorption of SCFA in the large intestine [32]. In the present study, the IDF content increased in the diet as supplementation of alfalfa meal was increased. However, it only led to significant increase of butyrate concentration in the caecal and colonical digesta, while acetate and propionate were not significantly affected among the four dietary treatments, which was inconsistent with previous study [8] regarding the effects of alfalfa meal on the SCFA concentrations in feces of growing pigs. The possible reason is that alfalfa meal mainly contains IDF, which means the limited capacity of fermentation to produce SCFA in the large intestine [33, 34]. Therefore, different intestinal segment has different SCFA concentrations. Another reason may stem from the different gut microbiota background existing in pigs used in different studies [35], which may also lead to the discrepancy with the previous study. However, the present result implied that moderate level of IDF (supplemental 10% alfalfa meal in the diet), increased microbial activity and butyrate concentrations in hindgut digesta of pigs, while low level (5% alfalfa meal) or higher level (15% alfalfa meal) of IDF containing diets have no effect on SCFA concentrations in hindgut digesta.

Many studies have documented that substantial soluble DF is fermented in the upper gastrointestinal gut (GIT) of the pigs, while the indigestible fiber (mainly insoluble) is fermented by the caecal microbiota [36, 37]. To our knowledge, the current study is the first to investigate the effects of alfalfa-derived IDF on caecal microbiota of growing pigs using Illumina MiSeq sequencing. Analysis of intestinal microbial diversity shows that compared with CAE0 group, Chao1 richness index of CAE15 group was significantly decreased, which was supposed to be related to the decrease of phyla Proteobacteria, Actinobacteria, Gemmatimonadetes and Chloroflexi in CAE15 group. In addition, a large amount of SCFA generated from fiber fermentation could lead to the decrease of pH value in the hindgut, which may also decrease the number of certain pathogenic bacteria [38, 39]. The significant increase of evenness index Alatalo of CAE15 group indicated that the addition of alfalfa meal could effectively improve the evenness of the distribution of various bacteria, and thus formed a more balanced microecosystem in the caecum. However, richness index Chao1 of CAE15 group was significantly decreased. These inverse variations regarding evenness and richness index of caecal microbiota may partly account for the fact that no significant alteration in diversity index Shannon and Simpson existed in caecal microbiota between CAE0 and CAE15 group. The present results showed that compared with CAE0 group, feeding 15% dietary alfalfa meal led to significant decreased abundance of *Turicibacter*, *Acidiphilium*, *Paracoccus*, *Propionibacterium*, *Corynebacterium*, *Pseudomonas*, *Acinetobacter*, *Staphylococcus*, but significantly increased abundance of *Lachnospira*, *Marvinbryantia*, *Desulfovibrio*. Previous studies have reported that genera *Acidiphilium*, *Paracoccus*, *Pseudomonas*, and *Acinetobacter* all belong to Proteobacteria whose mark is a disease-causing and inflammation-associated phylum, most species of these genera are potentially pathogenic and can cause diseases for human and animal [40]. *Corynebacterium*, covers hundreds of species that most of which are pathotypes, was originally proposed for the causative organism of diphtheria. *Corynebacterium diphtheria* was defined as the typical species of this genus [41]. Zhang et al. [42] also found that alfalfa-containing diet significantly decreased the potential pathogen *Streptococcus suis* in the caecum and distal colon of piglets, which supported our viewpoint that the alfalfa meal inclusion can decrease pathogens, and thus benefit the gut health.

The genera *Lachnospira* and *Marvinbryantia* belong to the same family of the *Lachnospiraceae*, which is classified into *Clostridium* cluster XIVa, and positively correlated with intestinal epithelial cell energy metabolism and butyrate production [43, 44]. Previous studies showed that the decrease of the butyrate-producing related family *Lachnospiraceae* in the gastrointestinal microbiota is associated with the susceptibility of colorectal cancer, ulcerative colitis and diabetes in humans [45–47]. The phylogenetic analysis revealed that strains related to *Oscillibacter*, a member of the family *Ruminococcaceae* (belongs to *Clostridium* cluster IV), is suggested as a health-promoting bacterium and is applied to butyrate production [44, 48]. As a result, the alfalfa-derived IDF was primarily fermented into butyrate by the bacteria belonging to *Clostridium* cluster IV and XIVa, which is in agreement with the current result that alfalfa meal significantly increased the concentration of butyrate in the caecum of pigs. *Alloprevotella* and *Prevotella* are subordinate to the family of *Prevotellaceae*, the species of which have the common characteristics that produce acetate, succinate and small amounts of propionate as the major end-products. Species belonging to *Alloprevotella* and *Prevotella* also have the capacity to degrade fiber polysaccharides such as cellulose and xylans that is contained in the alfalfa meal [3, 7, 49, 50]. Our results was consistent with the study that compared the fecal microbiota of Burkina Faso (an African village) children who consumes a high-fiber diet with Europe children who consumes a typical western diet, in which genera *Prevotella* are preponderant and exclusive to the Burkina Faso children, which is related to cellulose hydrolysis [51]. Our study proved that alfalfa-containing diets (with high IDF) could decrease the number of certain pathogenic bacteria and increase the number of bacteria related to alfalfa fiber hydrolysis and butyrate production, which effectively formed a more balanced microecosystem in the caecum and demonstrate that alfalfa-containing diets are beneficial to swine gut health.

Due to the significant variation in the caecal bacterial community and significant increase in butyrate concentration of pigs fed high level of alfalfa meal, the mRNA expression of the relevant genes was quantified. We observed the mRNA expressions of *FFAR3*, *SMCT1*, *MCT1*, *GCG* and *PYY* were significantly increased in the caecum of pigs fed 15% dietary alfalfa meal. Meanwhile, the ADFI of pigs fed alfalfa meal has a tendency to decrease, which may be due to high-fiber content of diets. The mechanism is considered to be that SCFA fermented as a byproduct by DF increase gut hormones like PYY and GLP-1 secreted by enteroendocrine cells [16–18]. Our results showed that pigs offered alfalfa meal had higher butyrate concentration in the caecal digesta compared with the control group. In agreement with the significant increase of butyrate concentration, the expression of *MCT1* and *SMCT1* was up-regulated in pigs offered alfalfa meal in the present study. Moreover, a higher expression of SCFA receptor *FFAR3* was found in the caecal mucosa of pigs fed alfalfa meal, which was supported by previous studies that FFAR2 has affinity to acetate while FFAR3 can be activated by butyrate [14, 17]. Previous studies have proposed that the activation of FFAR3 can trigger the production and secretion of GLP-1 and PYY by enteroendocrine cells, which is confirmed in our results that the up-regulated mRNA expression of *PYY* and *GCG* (precursor of GLP-1) [9].

## Conclusions

The present study showed that IDF from alfalfa meal increased caecal butyrate-producing bacteria that led to the largely increase of butyrate concentrations, and subsequently activated mRNA expression of butyrate-affiliative gene *FFAR3*, as well as SCFA uptake and gut hormone genes in the caecal musosa of pigs.

## Additional file

Additional file 1: **Table S1.** Nutrient composition and non-starch polysaccharides (NSP) contents of alfalfa meal. **Table S2.** Primer sequences used for quantitative real-time PCR. (DOCX 16 kb)

## Abbreviations

ADF: Acid detergent fiber
ADFI: Average daily feed intake
ADG: Average daily gain
BW: Body weight
CP: Crude protein
DF: Dietary fiber
DM: Dry matter
FFAR: Free fatty acid receptor
G:F: Gain:feed
GCG: Proglucagon
GIT: Gastrointestinal tract
GLP-1: Glucagon-like peptide 1
IF: Insoluble fiber
MCT1: Monocarboxylate transpoter 1
NDF: Neutral detergent Fiber
OTUs: Operational taxonomic units
PCA: Principal component analysis
PYY: Peptide YY
SCFA: Short-chain fatty acids
SMCT1: Sodium-coupled monocarboxylate transporter 1
