# Supplementary material for: Alfalfa-containing diets alter luminal microbiota structure and short chain fatty acid sensing in the caecal mucosa of pigs
Source: J Anim Sci Biotechnol. 2018 Jan 9;9:11. doi: 10.1186/s40104-017-0216-y (PMC5769528; doi:10.1186/s40104-017-0216-y)
Supplement: Additional file 1: — Table S1. Nutrient composition and non-starch polysaccharides (NSP) contents of alfalfa meal. Table S2. Primer sequences used for quantitative real-time PCR. (DOCX 16 kb) [file 40104_2017_216_MOESM1_ESM.docx]

**Table S1**. Nutrient composition and non-starch polysaccharides (NSP) contents of alfalfa meal.

|  | Alfalfa meal |
| --- | --- |
| Nutrient composition, % | |
| Dry matter | 91.3% |
| Crude protein | 16.5% |
| Neutral detergent fiber | 50.6% |
| Acid detergent fiber | 34.3% |
| Crude ash | 12.5% |
| NSP contents, g/kg dry matter | |
| Soluble NSP | 63.37 |
| Rhamnose | 0.87 |
| Fucose | 0.75 |
| Arabinose | 0.80 |
| Xylose | 0.06 |
| Mannose | 3.30 |
| Galactose | 2.06 |
| Glucose | 1.23 |
| Uronic acids | 54.30 |
| Insoluble NSP | 549.56 |
| Rhamnose | 8.06 |
| Fucose | 1.32 |
| Arabinose | 23.74 |
| Xylose | 101.92 |
| Mannose | 16.73 |
| Galactose | 14.39 |
| Glucose | 359.20 |
| Uronic acids | 24.20 |
| Total NSP | 612.93 |
| Klason lignin | 138.52 |
| Soluble dietary fiber | 63.37 |
| Insoluble dietary fiber | 688.08 |
| Dietary fiber | 751.45 |

**Table S2**. Primer sequences used for quantitative real-time PCR

| Gene symbol | Primer sequence | Product, bp |
| --- | --- | --- |
| β-actin | F: TGCGGGACATCAAGGAGAAG  R: AGTTGAAGGTGGTCTCGTGG | 216 |
| FFAR2 | F: CGTGTTCATCGTTCAGTA  R: GAAGTTCTCATAGCAGGTA | 76 |
| FFAR3 | F: TGGAGACCTTACGTGTTG  R: CGAGGATGAGAAGTAGTAGAT | 75 |
| SMCT1 | F: CGCAGATTCCTACTAACC  R: GATTGTCAGTTCCACCAT | 114 |
| MCT1 | F: CATCAACTACCGACTTCTG  R: TACTGGTCTCCTCCTCTT | 79 |
| MCT4 | F: TGCAGTGTGTGTGTGAATCG  R: GTTGAGCATGATGAGCGAGG | 183 |
| MCT5 | F: TTGAGTCAGTCGGTCAGCTT  R: CCACCGGAGAAAATGGCAAA | 178 |
| GCG | F: CAAGAGGAACAAGAATAACAT  R: AAGAACTTACATCACTGGTA | 87 |
| PYY | F: AGATATGCTAATACACCGAT  R: CCAAACCCTTCTCAGATG | 93 |
